# Hybrid Electrospun Conductive Nanofibers for Emerging Organic Contaminants’ Degradation in Visible Light Photocatalysis: A Review

**DOI:** 10.3390/ijms26189055

**Published:** 2025-09-17

**Authors:** Maria Râpă, Badriyah Alhalaili, Florin Aurel Dincă, Andra Mihaela Predescu, Ecaterina Matei, Ruxandra Vidu

**Affiliations:** 1 Department of Metallic Materials Processing and Environment Engineering, Faculty of Material Science and Engineering, National University of Science and Technology Politehnica Bucharest, 313 Splaiul Independentei, 060042 Bucharest, Romania; maria.rapa@upb.ro (M.R.); andra.predescu@upb.ro (A.M.P.); ecaterina.matei@upb.ro (E.M.); 2 Nanotechnology and Advanced Materials Program, Kuwait Institute for Scientific Research, P.O. Box 24885, Safat 13109, Kuwait; bhalaili@kisr.edu.kw; 3 American Romanian Academy of Arts and Sciences, P.O. Box 2761, Citrus Heights, CA 95611-2761, USA; 4 Biotechnical Systems Engineering Doctoral School, National University of Science and Technology Politehnica Bucharest, 313 Splaiul Independentei, 060042 Bucharest, Romania; florin_aurel.dinca@stud.isb.upb.ro

**Keywords:** conductive polymeric nanofibers, photocatalysts, organic pollutants, degradation

## Abstract

Emerging organic contaminants (EOCs), including polychlorinated bisphenyls (PCBs), pharmaceuticals, personal care products, pesticides, polycyclic aromatic hydrocarbons (PAH), and dyes, are among the most hazardous pollutants found in water bodies and sediments. These substances pose serious threats to the environment and human health due to their high toxicity, long-range mobility, and bioaccumulation potential. Although various methods for degradation of organic pollutants exist, photocatalysis using ultraviolet (UV) and visible light (VIS) has emerged as a promising approach. However, its practical applications remain limited due to challenges such as the use of powdered photocatalysts, which complicates their removal and recycling in industrial settings, and the restricted solar availability of UV light (~4% of the solar spectrum). This review investigates the effectiveness of hybrid electrospun conductive polymer nanofibers on metal oxide photocatalysts such as TiO_2_ and ZnO (including doped and co-doped forms) and fabricated via mono- or coaxial electrospinning, in the degradation of EOCs in water under visible light. Furthermore, strategies to enhance the fabrication of these hybrid electrospun conductive nanofibers as visible-light-responsive photocatalysts, such as the inclusion of dopants and/or plasmonic materials, are discussed. Finally, the current challenges and future research directions related to electrospun nanofibers combined with photocatalysts for the degradation of EOCs in water treatment applications are outlined.

## 1. Introduction

Emerging organic contaminants (EOCs) such as polychlorinated biphenyls (PCBs), polycyclic aromatic hydrocarbons (PAHs), pharmaceuticals, personal care products (PCPs), pesticides, dyes, endocrine-disrupting chemicals (EDCs), and microplastics (MPs) are among the most hazardous classes of pollutants found in water bodies and sediments [1,2,3,4,5,6,7,8,9]. EOCs result from anthropogenic industrial, agricultural, and urban activities and animal husbandry and possess a special combination of physical and chemical properties such that, once released into the environment, they remain intact for very long periods of time [10,11]. The dyeing and printing industries are among the most significant sources of water pollution, releasing approximately 280,000 tons of dyes annually of the total dyes used into wastewater [12]. Carcinogenic dye effluents possess complex organic compositions and contain a wide range of pollutants that present significant risks to aquatic ecosystems and the surrounding environment and may affect the health of nearby plant, animal, and human communities [13,14,15]. The widespread use of pesticides in agriculture often results in aquatic environmental pollution due to soil leaching [16,17,18,19]. Since plants absorb only a fraction of the applied pesticides, the remainder is left unutilized and can migrate into surrounding ecosystems, ultimately contaminating surface and groundwater resources, reaching various food products and drinking water. For instance, the main intermediate breakdown products of diuron (DU), chemically known as N-(3,4-dichlorophenyl)-N,N-dimethyl-urea, include N′-(3,4-dichlorophenyl)-N-methylurea (DCPMU), 3,4-dichlorophenylurea (DCPU), 3,4-dichloroaniline (DCA), and N′-(3-chlorophenyl)-N-methylurea (mCPMU) compounds, considered highly hazardous to humans. The presence of antibiotics in the environment can promote the emergence and spread of antibiotic-resistant bacteria (ARB) and antibiotic resistance genes (ARGs), posing significant health risks to both humans and animals [20,21,22,23].

Common strategies for removing EOCs from the environment include adsorption [24,25,26,27,28], membrane technology [29,30,31], electrokinetic remediation [32], advanced oxidation processes (AOPs) [33,34], supercritical water oxidation [35,36,37], wet air oxidation [38], incineration [39,40], phytoremediation [41,42], and biofiltration and bioreactor methods [43,44]. These techniques are often limited by high operational costs, incomplete pollutant removal, and the potential for secondary contamination of water bodies [45,46]. For instance, antibiotics are not effectively removed by conventional biological treatment methods and often require extended degradation time due to their inherent antibacterial properties, which inhibit microbial activity [47,48]. Large-scale application of traditional methods for the elimination of EOCs has remained limited.

Among them, photocatalysis, as an AOP, is considered a cutting-edge and environmentally friendly water treatment method capable of breaking down the complex chemical structure of organic pollutants. Photocatalysis enables faster degradation and eventual mineralization under optimal conditions of degradation time, efficiency of the photocatalyst, catalyst dose, analyte concentration, solution pH, and type of light source [49]. The characteristics of an excellent photocatalyst should include nontoxicity, abundance, cost-effectiveness, and recyclability. In practical applications, no single material meets all these criteria. Metal oxides as photocatalysts face several limitations. TiO_2_ and ZnO show a wide band gap (~3.2 eV), which restricts light absorption to the ultraviolet region, which represents only 5% of solar light. Moreover, metal oxides suffer from a high rate of electron/hole (e^−^/h^+^) recombination, low quantum yield, or photocorrosion, which significantly reduce their overall photocatalytic performance in aqueous environments. Additionally, their dispersion in powder form presents significant challenges for post-treatment recovery. The separation of suspended nanoparticles (NPs) remains a critical bottleneck for the scalability of water remediation technologies. To address these challenges, considerable research efforts have been directed toward enhancing charge separation and modifying metal oxide photocatalysts to extend their photoresponse into the visible light (VIS) region [16,50]. Strategies such as doping with metal or non-metal elements, coupling with other semiconductors, and surface modification with plasmonic materials have shown potential in improving their photocatalytic performance under solar irradiation.

**Figure 1:**
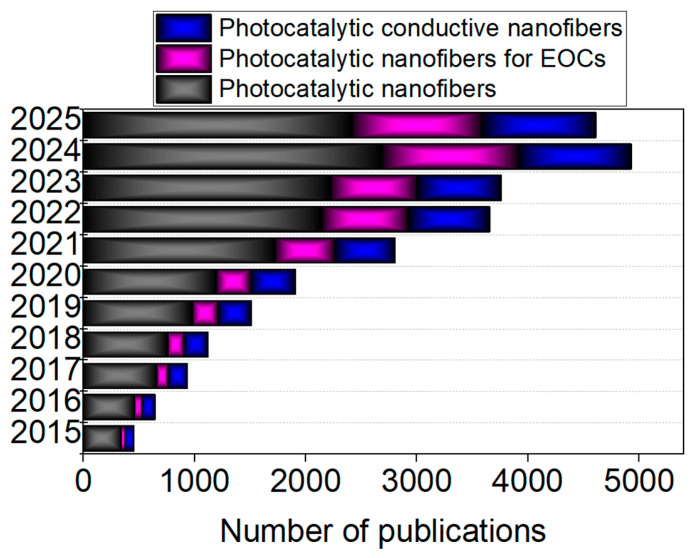
Published papers found by searching keywords “photocatalytic nanofiber”, “photocatalytic nanofibers for EOCs”, and “photocatalytic conductive nanofiber” in each year from 2015 to present based on ScienceDirect database.

Photocatalytic nanofibers represent a novel light-responsive catalytic platform, comprising functional catalyst particles immobilized on or integrated into a robust fiber-based scaffold that provides extensive surface exposure and a hierarchical pore network [16,51,52,53]. Researchers’ interest in photocatalytic nanofibers for removal of EOCs has grown in the last several years, as illustrated in Figure 1. This emerging class of materials effectively merges the strengths of active catalytic particles and fiber-based structures, delivering multiple key advantages: (1) outstanding photocatalytic activity; (2) functionality in mild reaction environments; (3) economical implementation; (4) convenient recyclability; (5) low potential for secondary contamination post-application; and (6) robust mechanical durability, capable of enduring physical stress, such as elongation, flexing, torsion, and folding [54]. Among various photocatalysts, hybrid electrospun nanofibers have gained prominence due to their large surface area, ease of functionalization, and enhanced charge separation efficiency, making them promising candidates for next-generation photocatalytic systems.

Hybrid electrospun nanofibers are typically fabricated by incorporating photocatalytic materials such as TiO_2_, ZnO, g-C_3_N_4_, or metal–organic frameworks (MOFs) into polymeric matrices like poly(vinyl alcohol) (PVA), poly(acrylonitrile) (PAN), poly(methylmethacrylate) (PMMA), or poly(styrene) (PS). The recent literature suggests that the conductive polymer layered semiconductor [50] and photoactive metal–organic frameworks (MOFs) [55,56] are highly promising candidates for the photocatalytic degradation of organic pollutants. The construction of heterojunction structures has emerged as a widely adopted strategy to improve the degradation performance of hybrid electrospun photocatalysts [57]. The development of step-scheme (S-scheme) heterojunctions has further advanced visible-light-driven photocatalysis by effectively utilizing solar energy.

This review outlines current strategies to prepare conductive photocatalytic fibers using synergic work of conductive polymers with oxide semiconductors for the degradation of EOCs under visible light. It also discusses challenges and future prospects of their applications, providing a comprehensive overview of emerging conductive photocatalytic fibers to support researchers in broadening their practical uses.

## 2. Hybrid Electrospun Conductive Nanofibers for Photocatalysis Under VIS Light

### 2.1. Conductive Polymer Semiconductors

**Figure 2:**
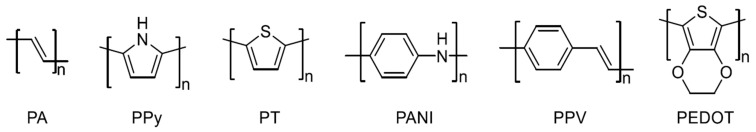
Chemical structures of representative CPs: poly(acetylene) (PA), poly(pyrrole) (PPy), poly(thiophene) (PT), poly(aniline) (PANI), poly(phenylene vinylene (PPV), and poly(3,4-ethylenedioxythiophene) (PEDOT) [59].

Conductive polymers (CPs) emerged in the mid-1970s as a distinctive class of macromolecular materials characterized by their intrinsic electrical conductivity, which is comparable to that of metals and conventional inorganic semiconductors [50,58,59]. Well-known representatives include polyacetylene (PA), polyaniline (PANI), polypyrrole (PPy), and polythiophene (PT), along with its derivatives, such as poly(3,4-ethylenedioxythiophene) (PEDOT) (Figure 2).

These materials exhibit a variety of advantageous properties, including tunable chemical and electrochemical behavior, lightweight nature, cost-effectiveness, outstanding compatibility with biological systems, and a reversible doping–dedoping process. Notably, their electrical conductivity can be precisely modulated over an extensive range (10^−11^ to 10^5^ S·cm^−1^) by applying suitable doping techniques. The sp^2^-hybridized conjugated backbone of conductive polymers is primarily responsible for their distinct electronic and optical properties. Upon oxidation (doping), the resulting resonance-stabilized delocalization of *π*-electrons and positively charged species (h^+^) facilitates efficient charge transport along the polymer chain. Owing to these attributes, conductive polymers are highly suitable for use as organic semiconductor materials with advanced applications in optoelectronics [60,61], chemical and biosensing [62,63,64], and photocatalytic applications for environmental pollution [50,65,66,67,68,69]. PANI, a *p*-type semiconductor, is recognized to exhibit a slow charge recombination rate through e^−^ transfer processes and a strong adsorption capacity for heavy metal ions/dyes because its molecular chains contain abundant amino/imino functional groups [70,71,72]. The existence of quinone and benzene groups with delocalized *π*-conjugated structures and electrochemical active sites in PANI makes it a good candidate for coupling with other semiconductors. Additionally, PANI and PPy function as effective sensitizers, broadening the photoactivity of TiO_2_ into the visible spectrum [73].

### 2.2. Conductive-Polymer-Integrated Photocatalysts

The selection of semiconductor photocatalysts, which have high performance, high stability, and easy recyclability, is critical for scientists. Common inorganic semiconductor photocatalysts include TiO_2_ (doped for visible light activity) [74,75,76,77], ZnO [16,66], carbon nanotubes (CNTs) [65], and Cu_2_O [10]. These photocatalysts exhibit several limitations, including a relatively small specific surface area, rapid recombination of photogenerated e^−^/h^+^ pairs, and restricted absorption within the visible light spectrum.

Overcoming the limitations of TiO_2_ nanoparticles, TiO_2_ nanofibers demonstrate significantly improved charge separation and transfer dynamics, along with enhanced suppression of e^−^/h^+^ recombination [78]. These improvements are primarily attributed to the three-dimensional interconnected network of TiO_2_ nanoparticles within the nanofiber architecture. Liu et al. [74] reported TiO_2_ nanofibers fabricated via electrospinning and subsequent calcination, which were further modified through vapor-phase polymerization to incorporate PEDOT. The resulting composite exhibited remarkable photocatalytic performance, achieving a 125% degradation efficiency under UV light for phenazopyridine (PAP), used as a model organic contaminant.

Similarly, ZnO nanocrystals were deposited onto a flexible mat of core–shell electrospun nanofibers composed of PAN nanofibers impregnated with PPy [66]. The heterojunction formed between PPy, a *p*-type conductive polymer, and ZnO, an n-type semiconductor, facilitates efficient separation of photogenerated charge carriers at the interface, thereby enhancing the photocatalytic degradation of persistent organic pollutants under UVA irradiation. Another paper reported a PAN/PU/β-CD@Ag nanofiber membrane based on ZnO NPs synthesized in situ via a hydrothermal process, resulting in excellent photocatalytic activity under visible light. This membrane achieved 71.5% degradation of methylene blue (MB) and 70.5% degradation of TCH [79].

Conductive-polymer-integrated photocatalysts have emerged as highly effective materials for breaking down organic contaminants and overcoming the limitations associated with powder photocatalysts, such as the need for separation from water. In a comparative study on the photocatalytic performance of a PANI–TiO_2_ nanocomposite versus TiO_2_ (Degussa P25) for the removal of Reactive Blue 19 (RB-19) dye, the PANI–TiO_2_ nanocomposite achieved complete degradation of RB-19 under acidic conditions, with a dye concentration of 50 mg/L and a catalyst dosage of 1 g/L [80]. Jiang et al. [81] proposed a PANI/TiO_2_-based composite in the form of a 3D hydrogel, which proved effective for removing organic contaminants and demonstrated ease of recyclability. Wang et al. [82] fabricated a CoW_11_Mn/PANI/TiO_2_ ternary composite through electrostatic self-assembly. The resulting photocatalyst efficiently degraded gentian violet (GV), reaching a degradation rate of 92.63% under ideal conditions. It also showed consistent performance with a negligible decline in catalytic activity after three consecutive uses. A three-dimensional (3D) network-structured composite of reduced GO–PANI/TiO_2_ was effectively fabricated by Cui et al. [83] through a two-step procedure that combined a hybridization step with a water bath technique. The resulting rGO–PANI/TiO_2_ hydrogels demonstrated complete (100%) removal of phenol through photoelectrocatalytic (PEC) treatment, greatly surpassing the efficiencies of individual photocatalytic (42%) and electrocatalytic (68%) processes. Alenizi et al. [84] reported almost complete photocatalytic degradation of Congo Red (CR) at concentrations of 10 mg/L and 20 mg/L within 120 and 180 min, respectively, using a g-C_3_N_4_/TiO_2_@PANI nanocomposite synthesized via an in situ chemical oxidative polymerization technique. Faisal et al. [85] developed a ternary photocatalyst consisting of PPy–carbon black (PPy-C) and platinum-NP-doped ZnO through an optimized sol–gel synthesis combined with a straightforward ultrasonication approach. The synthesized Pt@PPy-C/ZnO catalyst demonstrated 94.0% elimination of the linezolid antibiotic in just 40 min, showing a rapid degradation rate approximately 1.67 times greater than that of pristine ZnO. Yu et al. [10] constructed a *p-n* heterojunction between PANI and Cl-Cu_2_O using a bacterial cellulose (BC) film as support, which exhibited a photocatalytic degradation efficiency of 96.3% for oxytetracycline (OTC) under visible light irradiation. The improved performance was attributed to the internal electric field formed at the *p*–*n* heterojunction, which significantly facilitates the separation of photogenerated e^−^/h^+^ pairs. Similarly, Zhou et al. [86] developed a PPy@(BC/g-C_3_N_4_) photocatalytic membrane by incorporating PPy onto a BC carrier. Under low-power xenon lamp irradiation (λ > 420 nm), the modified membrane achieved a 64.28% degradation efficiency for tetracycline hydrochloride (TCH) within 2 h, which was 5.27 times higher than that of unmodified BC/g-C_3_N_4_ (12.20%). Additionally, the PPy@(BC/g-C_3_N_4_) membrane demonstrated excellent reusability, maintaining over 80% of its original catalytic activity after 10 reuse cycles. Converting ZnO to zinc oxide nanorods (ZnRs) increases the surface area, while incorporating carbon materials such as graphene, carbon nanotubes, or amorphous carbon can significantly improve photocatalytic performance. Anirudhan et al. [16] synthesized a stable ZnR@CGR/PANI photocatalyst that maintained its activity for up to five cycles, containing ZnR, carboxylic graphene (CGR), and PANI, which demonstrated 99.0% adsorption and photodegradation of the pesticide diuron (DU) from aqueous solutions at pH 2.5 for 90 min under visible light irradiation. Asadpoor et al. [87] developed a Ni-ZnO/Bi_2_WO_6_/PANI ternary heterojunction photocatalyst via an in situ hydrothermal method. Under optimal conditions, the nanocomposite achieved complete removal of ciprofloxacin (CIP) within 70 min of visible light irradiation, demonstrating excellent photocatalytic performance.

The acid-etching technique was used to improve the specific surface area, facilitate charge transport, and mitigate the limited visible light absorption of g-C_3_N_4_ [11]. Kumar et al. [11] synthesized nanocomposites of acid-etched g-C_3_N_4_ nanosheets and PANI nanofibers using an in situ oxidative polymerization method. The resulting photocatalyst achieved a maximum degradation efficiency of 99.3% for methyl orange (MO) within 25 min and 96.3% for CR within 150 min.

Hybrid noble-metal-based conductive polymer nanostructures (CPNs) are particularly attractive as multimodal platforms for the simultaneous degradation and sensing of organic pollutants. By leveraging plasmonic photocatalysis and surface-enhanced Raman scattering (SERS), these materials offer strong electromagnetic enhancement [88]. Liu et al. [89] developed an effective strategy for the transfer of photoexcited charges in the photocatalytic heterojunction system by establishing a conductive interface between g-C_3_N_4_ and P_3_HT semiconductors by introducing Ag. The ideal interface should possess high compatibility with the semiconductor materials, appropriately matched work functions, and efficient charge transport capabilities. Ghosh et al. [88] synthesized Au/PEDOT nanohybrids that effectively decolorized MO and rhodamine B (Rh B) aqueous solutions under visible light irradiation. These nanohybrids exhibited excellent cycling stability and SERS recyclability in the detection of MO and Rh B solution at concentrations as low as 10^−6^ M, highlighting their potential for the simultaneous sensing and removal of organic pollutants.

ABO_3_ perovskite semiconductors such as BiFeO_3_ NPs and LaFeO_3_ are promising candidates for use as photocatalysts and Fenton catalysts in pollutant treatment due to their low cost, high Curie temperatures, favorable dielectric properties, narrow band gaps (2.1 eV/2.07 eV), and excellent chemical stability [90,91]. Additionally, as an emerging piezoelectric material, BiFeO_3_ can convert mechanical energy into electrical energy, generating an internal electric field that effectively suppresses charge-carrier recombination.

### 2.3. Fabrication of Hybrid Electrospun Conductive Nanofibers

Hybrid electrospun conductive nanofibers combine the benefits of electrospun polymers with electrical conductivity, making them suitable for water purification applications. Commonly employed strategies for their fabrication include electrospinning of conductive polymeric solutions to form nanofibers or core–shell structured fibers; post-electrospinning surface modification, where pre-formed nanofibers are coated with conductive materials; and vacuum self-assembly techniques to integrate conductive components into or onto nanofiber structures.

Table 1 summarizes the most important parameters of hybrid conductive composite fibers processed through the electrospinning technique and their efficiency in the degradation of EOCs.

#### 2.3.1. Electrospinning Process

Electrospinning is widely acknowledged as a simple, versatile, and cost-effective technique for producing continuous ultrathin fibers. This method offers scalable production, adjustable fiber architectures, high surface area, hierarchical porosity, and excellent structural flexibility, being involved in various applications [51,95,96,97,98,99,100,101,102,103,104]. Electrospinning has also emerged as a promising approach for reducing waste carbon-based materials and removing organic pollutants from water [105,106]. Recently, waste cigarette butts were repurposed to fabricate electrospun cellulose acetate (CA) nanofibrous membranes, which were subsequently modified with PANI through chemical oxidative polymerization [107]. These membranes were employed for the removal of MO and rhodamine chloride (RhC) dyes from aqueous solutions. The maximum equilibrium adsorption capacities reached 24.87 mg/g with a 99% removal efficiency for MO and 6.93 mg/g with a 55% removal rate for RhC. Notably, the membranes maintained their performance over up to seven adsorption–desorption cycles.

The production of photocatalytic fibers involves three straightforward steps: synthesizing the photocatalyst, preparing a homogeneous spinning solution containing both the photocatalyst and polymer, and finally, performing the electrospinning directly [51]. Blending a CP with a carrier polymer, even at very low concentrations, can form good nanofibers with surprisingly high conductivities [59].

The drawbacks of CPs for fabrication into nanofibers are their rigidity and low molecular weight. To address these limitations and enhance the charge-carrier mobility of electrospun CP fibers, various strategies have been developed. Conductive polymer nanofibers can be fabricated by electrospinning/co-electrospinning of conductive polymer solutions, by coating non-conductive polymer fibers with conductive materials, or by vacuum self-assembly [58,108].

Table 2 shows the specific characteristics, performance, and perspectives for development of hybrid electrospun conductive nanofibers designed for removal of EOCs.

Polyacrylonitrile (PAN) and SiO_2_ are well known as carriers for achieving poly(isothianaphthene) (PITN) [93] and PANI nanofibers [73], which demonstrated both a high surface-area-to-volume ratio and favorable recycling properties. The reduction of ~20% in the photocatalytic activity of PANI-coated TiO_2_/SiO_2_ nanofiber membranes after five recycling cycles was probably attributed to the presence of residual organic compounds of MO in the nanofibers, which blocked some active sites on the photocatalyst [73]. At elevated levels of the carrier polymer, several essential characteristics of the CPs, such as electrical conductivity and electrochemical activity, are reduced. For instance, polyethylene oxide (PEO) may be employed as an additive to improve electrical and ionic conduction, while also providing sufficient jet stability to support the formation of a solidified nanofiber mat [62].

**Figure 3:**
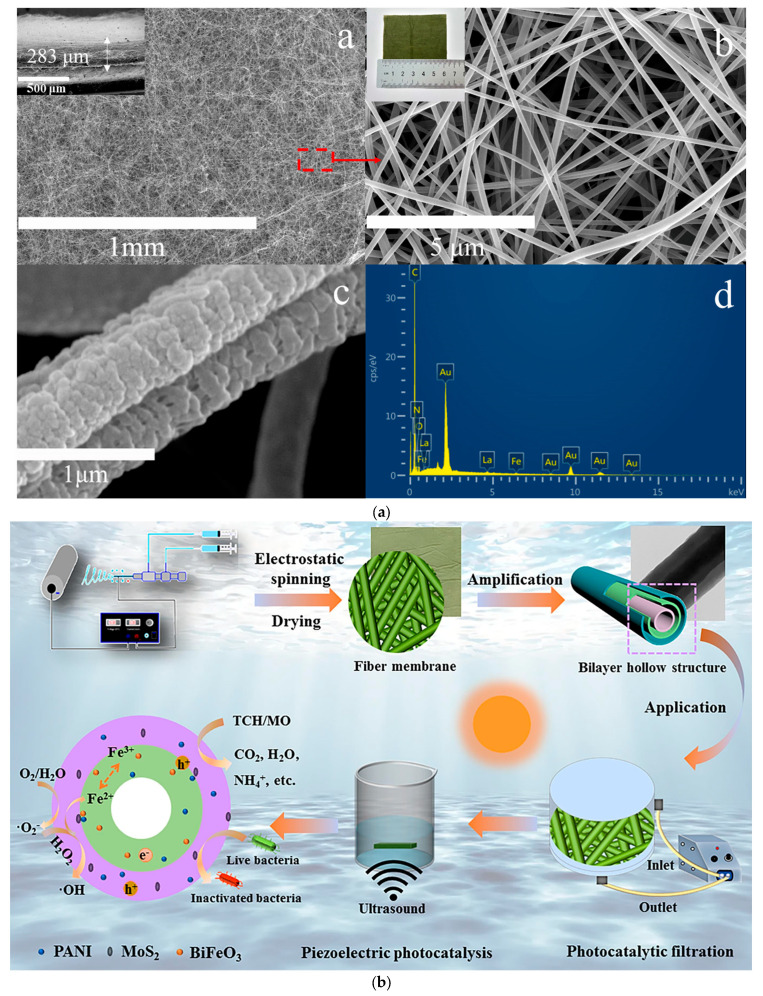

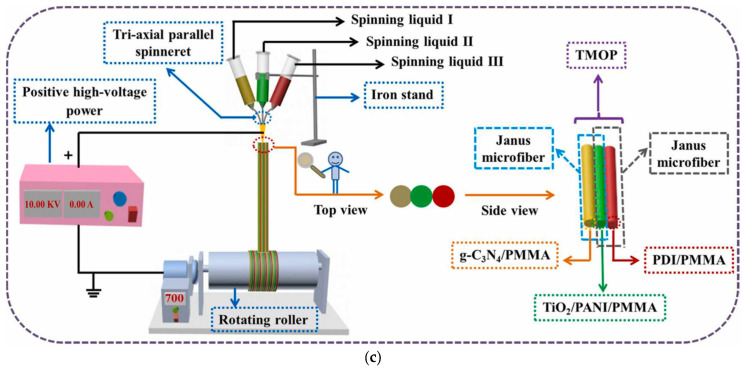
SEM and optical images and EDX of PC@PL membrane (**a**). Reproduced from [91] with permission from Elsevier. Multifunctional MoS_2_/PANI/PAN@BiFeO_3_ (PPBM-H) bilayer hollow nanofiber membrane achieved via coaxial electrospinning (**b**). Reproduced from [90] with permission from Elsevier. Self-supporting tricolor-typed microfiber oriented-heterostructure photocatalyst (TMOP) produced via triaxial electrospinning (**c**). Reproduced from [94] with permission from Elsevier.

Coaxial electrospinning can be used to produce core/shell nanofibers. Figure 3a–c illustrate coaxial and triaxial electrospinning techniques for fabricating g-C_3_N_4_/PAN/PANI@LaFeO_3_ (PC@PL) cable nanofiber membranes [91], innovative multifunctional MoS_2_/PANI/PAN@BiFeO_3_ (PPBM-H) bilayer hollow nanofiber membranes [90], and a self-supporting tricolor-typed microfiber oriented-heterostructure photocatalyst based on [g-C_3_N_4_/PMMA]/[TiO_2_/PANI/PMMA]/[self-assembled 3,4,9,10-perylene tetraformyl diimide (PDI)/PMMA] (TMOP) [94]. To prevent mixing of the three spinning liquids during the electrospinning process, it is essential that they have similar viscosities and flow rates. This ensures stable separation and uniform formation of distinct microfiber components. The PC@PL membrane exhibited a porous, disordered surface with an average pore size of 0.92 μm and a thickness of ~283 μm, as shown in Figure 3a. Scanning electron microscopy (SEM) images revealed a highly interconnected, rough nanofibrous structure, attributed to solvent evaporation, which exposes more functional groups of PANI. The membrane’s green color confirmed the incorporation of PANI. EDX analysis detected C, O, N, Fe, and La, confirming the successful integration of g-C_3_N_4_ and LaFeO_3_ via coaxial electrospinning [91].

#### 2.3.2. Post-Electrospinning Surface Modification

Various types of synthetic polymers have been used for fabricating electrospun conductive coatings, such as PAN, polyvinyl alcohol (PVA), polycaprolactone (PCL), polyvinylpirrolidone (PVP), and polystyrene (PS). PAN and PVP mats serve as excellent membrane supports for the in situ polymerization of CPs due to their high tensile strength, flexibility, and stability under elevated temperatures and acidic conditions. Polyarylene ether nitrile (PEN) is an emerging high-performance polymer characterized by exceptional thermal stability, chemical and oxidative resistance, robust mechanical strength, and compatibility with electrospinning processes [92]. The coating is also accomplished through dip-coating [65,73] or vapor-phase polymerization [74], producing a conductive shell around an insulating core.

**Figure 4:**
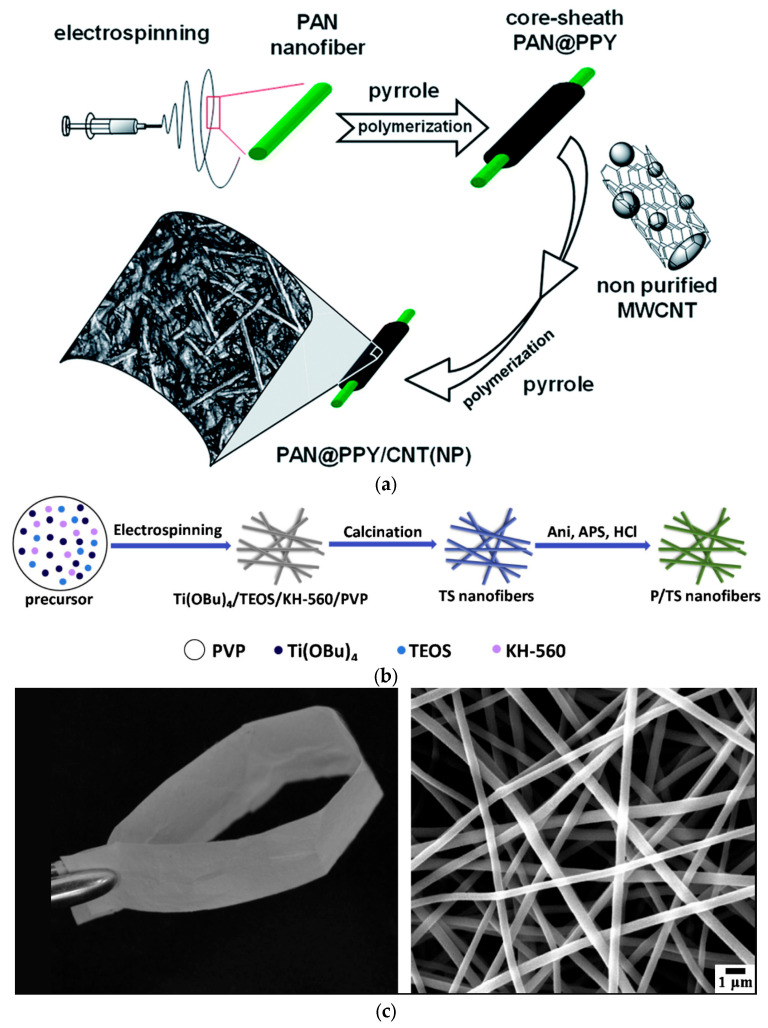
Synthesis of PAN@PPY-CNT(NP) composite (**a**) [65]; synthesis of PANI-coated TiO_2_/SiO_2_ (P/TS) membrane (**b**); flexible PANI-coated TiO_2_/SiO_2_ (P/TS) membrane and SEM image (**c**). Reproduced from [73] with permission from Elsevier.

Figure 4a,b show the preparation of PAN@PPY-CNT(NP) composite material [65] and PANI-coated TiO_2_/SiO_2_ (P-TS) nanofibrous membranes [73] using electrospinning, heat treatment, and in situ polymerization methods. To ensure complete wetting and penetration of the pyrrole polymerization solution into the PAN nanofibers, the mats were treated with a commercial UV–ozone system, which enhanced the hydrophilicity of PAN [65]. Subsequently, the PAN-PPY mats were impregnated with an aqueous dispersion of CNT(NP)s at a concentration of 200 ppm, followed by exposure to the PPY polymerization solution to improve their stability and mechanical resistance. To prevent the detaching of CNC(NT) from the fibers, the first and final PPY layers were necessary.

A strategy to improve the mechanical properties of PANI coated on a TiO_2_/SiO_2_ membrane was to mix TiO_2_ sol with TEOS and KH-560 (3-glycidyloxypropyltrimethoxysilane) [73]. Liu et al. [73] established that the TiO_2_/SiO_2_ (TS) nanofibers were fully coated with PANI after a reaction time of 4 h. When the amount of PANI exceeded a certain threshold, the excess PANI nanoparticles tended to form a thicker coating, which led to aggregation on the surface of the TS nanofibers. This can impede the transfer of photoexcited e^−^ from the outer PANI layer to the underlying TS nanofibers, affecting the number of radicals involved in the photodegradation of the pollutant. Flexible TS nanofiber membranes were easily bent, probably due to the presence of SiO_2_, and showed a uniform diameter and smooth surface, as is illustrated in Figure 4c.

Enhanced mechanical properties combined with a 49.43% degradation rate of MO were reported in the case of TiO_2_–PANI/thermoplastic poly(urethane) (TPU) elastic membranes [109].

Superhydrophilic membranes for oil-in-water (O/W) separation were fabricated by depositing PANI onto electrospun PAN nanofibers via in situ polymerization of aniline [110].

Bhadra et al. [111] prepared a PS/PANI-Ag membrane by coating PS nanofibers with PANI and Ag, which had 85% degradation efficiency for Azocarmine G dye solution under UV irradiation within 180 min. Additionally, the photocatalyst exhibited stronger antibacterial activity against Gram-negative bacterial strains compared to Gram-positive ones due to the presence of Ag.

The efficient and straightforward removal of organic pollutants, along with the mitigation of membrane fouling in aquatic environments, is vital and can be achieved through the use of engineered membranes with combined adsorption, filtration, and photodegradation functionalities. The spontaneous adsorption process coupled with photocatalysis is a promising method for reducing various contaminants in water [50]. A composite photocatalyst fabricated from the coating of PANI with TiO_2_ NPs improved the degradation of contaminants like 2,4-dichlorophenoxyacetic acid (2,4-D) and triclopyr acid (TCP) from water [50]. Thus, a photocatalyst concentration of 0.5 g L^−1^ under irradiation with a 125 W high-pressure mercury lamp for 150 min led to optimal removal efficiencies achieved at specific pH values: approximately 90.72% for TCP at pH 4 and 99.91% for 2,4-D at pH 5. This hybrid catalyst demonstrated remarkable activity for three cycles of photodegradation [50]. A composite membrane adsorption system based on poly(urethane) (PU) coated with PANI in a PVA solution was developed by Yasir et al. [9] for the enhanced removal of 17α-ethinylestradiol (EE2), a potent synthetic estrogen, from water. In batch adsorption experiments, 20 mg of the composite adsorbent was tested with 100 mL of hormone solution with a concentration of 0.20 mg/L for 3.5 h at 150 rpm, room temperature, and pH 7. The results indicated a removal efficiency of 90.33% and an adsorption capacity of 0.998 mg/g, respectively.

While surface coating is an effective method to impart conductivity, it may disrupt the desirable porous architecture of the nanofiber mats and negatively impact critical properties, such as biocompatibility, permeability, or mechanical flexibility.

#### 2.3.3. Vacuum Self-Assembly

**Figure 5:**
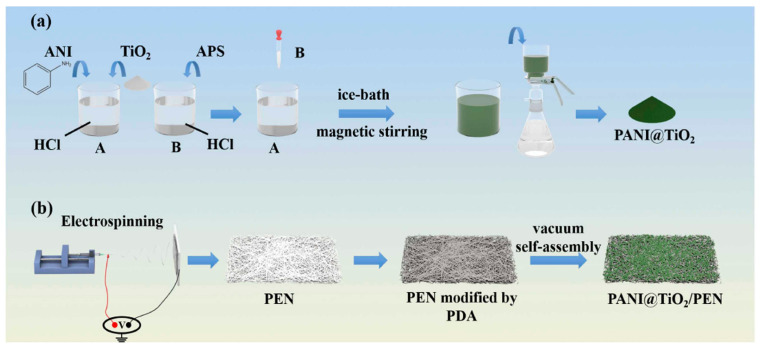
The synthesis of PANI@TiO_2_ composites via a vacuum-assisted self-assembly method (**a**); fabrication of the PANI@TiO_2_ nanofibrous membrane via electrospinning (**b**). Reproduced from [92] with permission from Elsevier.

Although vacuum self-assembly shares certain similarities with conventional coating methods, such as thin-film deposition, it is distinguished by its capacity to facilitate the spontaneous and often more ordered arrangement of nanostructures. This process is driven by interfacial forces and controlled solvent evaporation under vacuum conditions, resulting in enhanced structural uniformity and functional integration. In environmental nanotechnology, recent advances have shown that coupling photocatalytic degradation with interfacial solar-driven water evaporation can provide a dual-function strategy that simultaneously enables contaminant removal and water harvesting. Building on this concept, Li et al. [92] developed a robust electrospun nanofibrous superhydrophilic membrane, wherein PANI@TiO_2_ composites were uniformly anchored onto a high-performance PEN nanofibrous substrate via polydopamine (PDA)-mediated adhesion through a vacuum-assisted self-assembly method. PANI@TiO_2_ nanocomposites were synthesized by polymerization of aniline (ANI) in HCl, dispersion of TiO_2_ NPs via ultrasonic stirring (solution A), and addition of ammonium persulfate (APS) dissolved in HCl (solution B), as an initiator (Figure 5a,b). Notably, the plentiful hydroxyl and amine groups present in PDA molecules were introduced onto the PANI@TiO_2_ nanofibrous membrane via a vacuum-assisted self-assembly method, imparting superhydrophilicity to the membrane and enabling efficient water transport to support rapid evaporation. In situ polymerization of PANI on the TiO_2_ surface led to agglomeration. For the water evaporation test, Petri dishes insulated with polystyrene foam (PS) on both the inside and outside were used, ensuring that the light source irradiated only the membrane.

A concentration of 20 mg·mL^−1^ PANI@TiO_2_ in water suspension exhibited a maximum average evaporation rate of 3.23 kg·m^−2^·h^−1^ [92].

## 3. Photocatalytic Degradation Mechanism

### 3.1. Type-II Heterojunction Structure

Researchers are actively developing high-performance photocatalytic conductive membranes for the efficient removal of organic contaminants from water, aiming for materials that are easily recoverable and possess multifunctional properties. One promising strategy involves the fabrication of hybrid structures using electrospun conductive nanofibers, which enable the integration of photocatalytic and conductive functionalities. In this context, a Type-II heterojunction structure in electrospun nanofibers describes a system in which a donor and an acceptor material form a staggered band alignment, causing the spatial separation of e^−^ and h^+^ across the interface of different fiber components. This band alignment promotes efficient charge separation and reduced recombination, making it ideal for enhanced photocatalytic performance. The photocatalytic degradation of RB using the PAN@PPY–CNT(NP) photocatalyst was driven by CNT(NP)s, which served as sources of charge carriers and consequently facilitated the production of ·OH radicals under UV–Vis irradiation [65].

**Figure 6:**
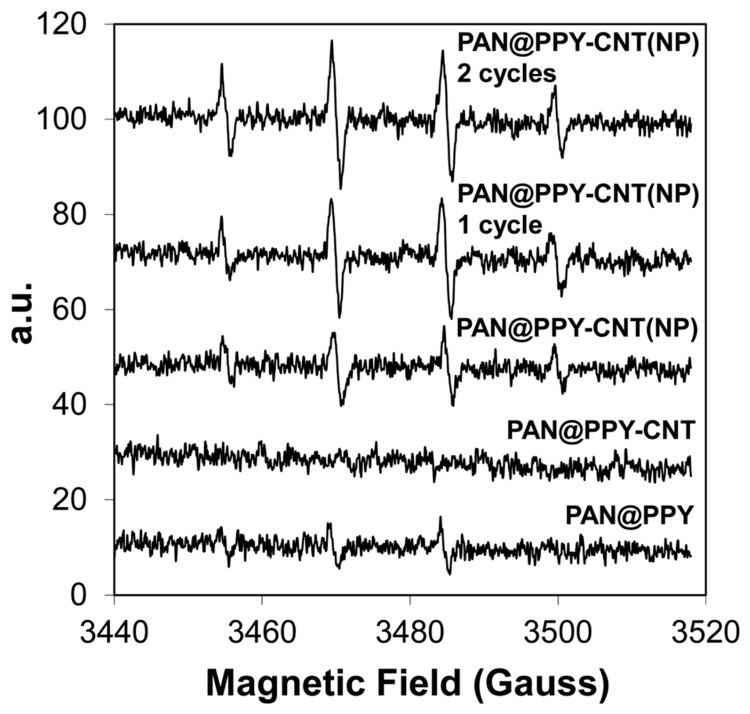
EPR spectra of PAN@PPY, PAN@PPY–CNTHCl, and PAN@PPY–CNT(NP) mats [65].

Electron Paramagnetic Resonance (EPR) analysis revealed that the suppression of ·OH radical generation was attributable to the PPy coating, which acted as a barrier to the incident light—Figure 6. The PAN@PPY–CNT(NP) samples were evaluated immediately after synthesis, after the first cycle of RB removal experiments, and subsequently after the second cycle.

It was reported that the metal oxide NPs embedded in CNT(NP)s played a key role in the PAN@PPY–CNT(NP) photocatalyst for the photocatalytic degradation of MB, while the adsorption of MB molecules occurred preferentially onto the PPY sheath [65]. However, the PAN@PPY–CNT(NP) photocatalyst was not effective for the removal of naphthalene and MO. Another finding is that its performance was lower compared with the TiO_2_-based composite benchmark. The loss of removal efficiency between the first and next cycles, as well as the stable efficiency observed during later cycles, was attributed to the washing step, which removed a large fraction of the compounds, except those irreversibly adsorbed on the most active PPY sites.

After one week of irradiation, the ultimate tensile strength, tensile modulus, and elongation at break decreased by approximately 60%, 50%, and 40%, respectively, compared to their initial values. In this PPy–CNT(NP) Type-II heterojunction, PPy acted as the electron donor while the CNTs served as the electron acceptor, thereby enhancing the charge separation and improving photocatalytic activity [65].

**Figure 7:**
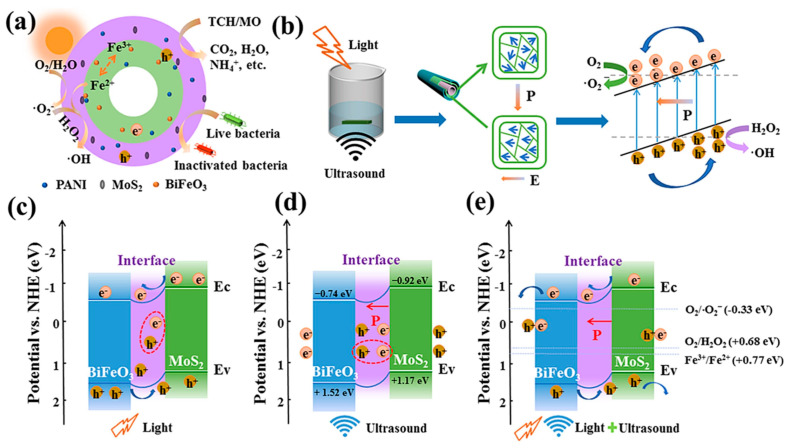
Photocatalytic degradation mechanism of MoS_2_/PANI/PAN@BiFeO_3_ (PPBM-H) in filtration systems (**a**); band structures of polarized BiFeO_3_ and BiFeO_3_/MoS_2_ under visible light and ultrasound (**b**); Type-II heterojunction structure of MoS_2_/PANI/PAN@BiFeO_3_ (PPBM-H) membrane (**c**); electrochemical potential measured relative to NHE during ultrasonic excitation (**d**); electrochemical potential measured relative to NHE under dual stimulation by light and ultrasound (**e**). Reproduced from [90] with permission from Elsevier.

Photocatalysis alone is often limited by the inevitable charge recombination at the heterojunctions, while piezocatalysis alone may suffer from reduced activity due to band bending and inefficient carrier dynamics. To overcome these limitations, Lin et al. [90] successfully developed a MoS_2_/PANI/PAN@BiFeO_3_ filtration membrane by the synergistic integration of piezocatalysis and photocatalysis techniques (Figure 7).

**Figure 8:**
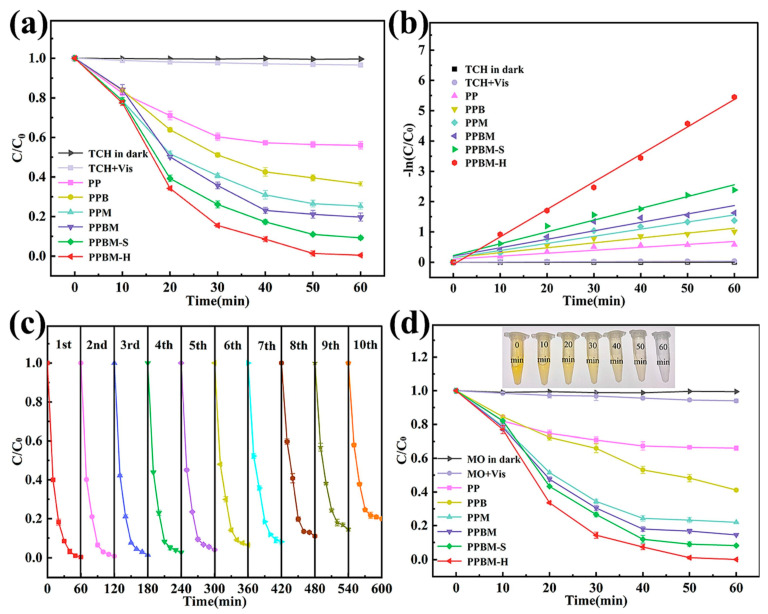
Photocatalytic filtration degradation of TCH under visible light (**a**); linear fitting of quasi-first-order kinetics (**b**); cyclic stability of PPBM-H under photo-filtration (**c**); photocatalytic filtration degradation of MO under visible light (**d**). PP = PANI/PAN nanofiber membrane; PN = PAN nanofiber membrane; PPB = PANI/PAN/BiFeO_3_ nanofiber membrane; PPM = PANI/PAN/MoS_2_ nanofiber membrane; PPBM = PANI/PAN/BiFeO_3_/MoS_2_ hybrid monolayer nanofiber membrane; PPMS = PANI/PAN/BiFeO_3_/MoS_2_ bilayer solid nanofiber membrane. Reproduced from [90] with permission from Elsevier.

Figure 8a shows the excellent performance of the PPBM-H photocatalyst used for the degradation of TCH (99.8%) after 60 min under visible light irradiation, compared with PPB and PPM, which achieved removal rates of 63.5% and 74.7%, respectively. Figure 8b–d show the removal efficiency of PPBM (80.3%), the high degradation kinetic constants of PPBM-H, the photocatalytic filtration performance (which remained around 80% after ten cycles), and the degradation profile for MO [90].

This hybrid electrospun system leveraged the advantages of both processes, facilitating both improved charge separation and energy conversion efficiency for the degradation of THC and MO pollutants. Under ultrasonic excitation, BiFeO_3_ in the PPBM-H nanofiber membrane underwent a domain alignment and generated a piezoelectric effect and internal electric field (P), which enhanced the charge separation. When combined with visible light, this reduced e^−^/h^+^ recombination and increased the formation of the reactive radicals, achieving 99.5% and 99.9% removal for TCH and MO, respectively, from wastewater within 60 min. Also, complete disinfection of *Escherichia coli* within 60 min was reported [90].

In another experiment, the photocatalytic degradation of MB by a PANI@TiO_2_/PEN nanofibrous membrane also followed a Type-II mechanism [92]. Under visible light, the photogenerated e^−^ from PANI were transferred to the CB of TiO_2_, while holes from TiO_2_’s valence band migrated to the HOMO of PANI. These charge carriers reacted with O_2_ and H_2_O/OH^−^ to form reactive oxygen species (ROS), such as ·O_2_^−^ and ·OH, which break down the dye.

**Figure 9:**
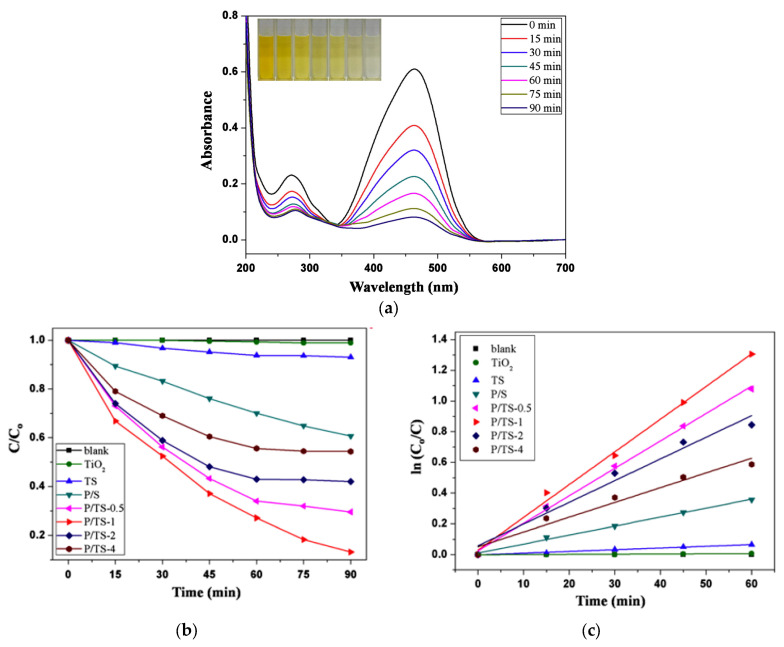
UV–vis absorption spectra of the MO solution with P/TS monitored over 1 h of reaction. The insets show digital photographs of the MO solutions taken at the same time as the UV–vis measurements (**a**), MO photocatalytic degradation (**b**), and linear kinetic analysis for TiO_2_ powder, TS, P/S, and P/TS nanofibers at various polymerization times (**c**) (P = PANI, T = TiO_2_, S = SiO_2_). Reproduced from [73,90] with permission from Elsevier.

A similar photocatalytic mechanism was observed for the PANI-coated TiO_2_/SiO_2_ nanofiber flexible membranes (P/TS) developed by Liu et al. [73]. The membranes were immersed in 3 mL of MO solution (1.5 mg L^−1^) and stored in the dark for 2 h to achieve the adsorption–desorption equilibrium. The photocatalytic degradation of MO was 87% under light irradiation [73]. The excellent photoactivity of the P/TS membrane was likely due to the synergistic interaction between TiO_2_ and PANI, which promoted improved charge separation and visible light absorption. Absorbance at 465 nm was used to quantify MO degradation. Photocatalytic efficiency under visible light irradiation followed the trend P/TS-1 > P/TS-0.5 > P/TS-2 > P/TS-4 > P/S > TS > TiO_2_ > blank, with P/TS-1 achieving 87% MO removal in 90 min—Figure 9a,b.

The degradation efficiency of MO declined slightly from 87% to 70% after five cycles; nevertheless, the membrane demonstrated robust photocatalytic stability. This decline was likely caused by the accumulation of residual dye molecules within the nanofibers, which may have blocked the active sites and hindered photocatalytic performance.

**Figure 10:**
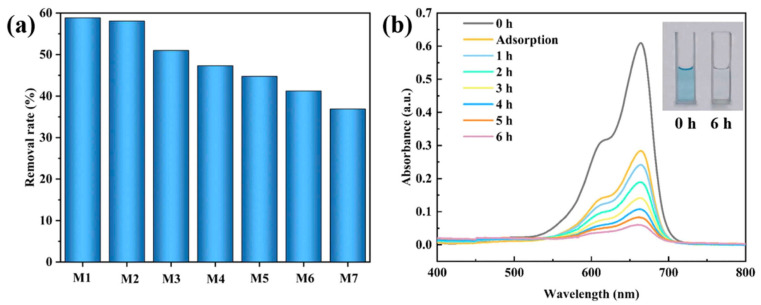
The removal rate of MB for PANI@TiO_2_/PEN composite nanofibrous membrane, where M1–M7 signifies concentrations of the PANI@TiO_2_ water suspension from 5 to 100 mg L^−1^ (**a**); the degradation efficiency of MB by M3 under sunlight irradiation (**b**) [92].

Under visible light irradiation, PANI acted as a photosensitizer for TiO_2_. The excited electrons in the PANI migrated to the CB of TiO_2_, while the holes (h^+^) from the valence band (VB) of TiO_2_ were transferred to the HOMO of PANI. During the reaction of the photogenerated electrons with O_2_, superoxide radicals (·O_2_^−^) were generated, and h^+^ reacted with H_2_O to produce ·OH, thus generating reactive oxygen species (ROS) that degraded the MO dye. This process promoted rapid separation of the photogenerated charges and slowed charge recombination, thereby significantly enhancing the photocatalytic performance of the prepared P/TS photocatalysts [73]. A similar mechanism was encountered in the case of a PANI@TiO_2_/PEN composite nanofibrous membrane, which degraded MB—Figure 10 [92].

### 3.2. Z-Scheme Heterojunction Structure

The Z-scheme heterojunction structure represents an advanced photocatalytic architecture. Unlike conventional Type-II heterojunctions, which promote spatial separation of charge carriers at the expense of redox potential, Z-scheme systems are specifically designed to retain strong redox ability while also achieving efficient charge separation.

**Figure 11:**
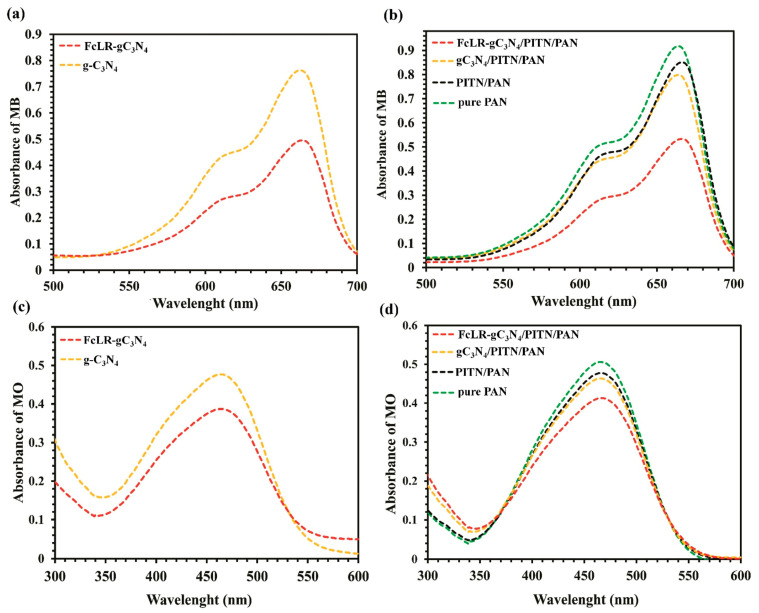
The photodegradation curves for MB in particle form (**a**) and fibrous form of photocatalysts (**b**). The photodegradation curves for MO in particle (**c**) and fibrous form of photocatalysts (**d**). Reproduced from [93] with permission from Elsevier.

The degradation efficiencies of MO and MB using photocatalysts in both particle and nanofiber forms were investigated. The results showed that the degradation efficiencies of MB and MO using photocatalyst particles were in the order of FcLR-gC_3_N_4_ > g-C_3_N_4_, and using photocatalysts in fibrous form, the order was FcLR-gC_3_N_4_/PITN/PAN > g-C_3_N_4_/PITN/PAN > PITN/PAN > pure PAN. When g-C_3_N_4_ was combined with FcLR, it exhibited higher photocatalytic activity than g-C_3_N_4_ alone, confirming the synergistic interaction between the conductive and photocatalytic components—Figure 11. This synergy facilitated more effective light absorption and better use of charge carriers, thereby accelerating the degradation of MB [93]. Also, the electrochemical impedance spectroscopy (EIS) and photocurrent measurements confirmed that the attachment of FcLR to g-C_3_N_4_ significantly improved the separation rate of photogenerated charge carriers, resulting in enhanced photocatalytic activity compared to g-C_3_N_4_ alone.

The optimal photocatalyst nanofiber dosage was 5 g. Increasing the temperature to 60 °C improved the degradation efficiency of MB from 92% to nearly 100% due to the enhanced dye–photocatalyst interactions and adsorption. However, the degradation efficiency declined with extended contact time, likely due to competition between intermediate degradation products and the dye for active sites on the photocatalyst. To clarify the degradation pathway of MB (92%) and MO (29%) dyes, Asgari et al. [93] proposed the following sequential photocatalytic mechanism: (1) excitation of charge carriers within the FcLR-g-C_3_N_4_/PITN/PAN composite under light exposure (Equation (1)), (2) generation of reactive oxygen species (ROS), primarily superoxide radicals (O_2_·^−^) (Equation (2)), and (3) oxidative degradation of dye molecules by O_2_·^−^ and other reactive intermediates formed in subsequent reactions (Equation (3)).

**Figure 12:**
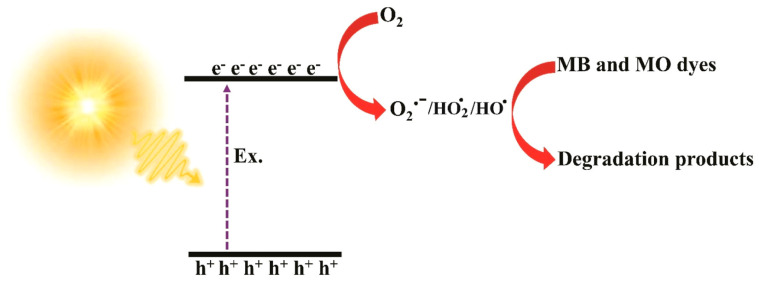
Mechanism of MB and MO dye photodegradation under visible light irradiation using the FcLR-g-C_3_N_4_/PITN/PAN ternary composite photocatalyst. Reproduced from [93] with permission from Elsevier.

Figure 12 presents a schematic illustration of the proposed photocatalytic mechanism for the degradation of dyes. Based on the reduction in the band gap from 2.6 eV in g-C_3_N_4_ to 1.7 eV in the composite, the Z-scheme heterojunction may be the most probable mechanism.

Photocatalyst + hυ → e^−^/h^+^ generation (1)

e^−^ + O_2_ → O_2_·^−^ (2)

O_2_·^−^ + H_2_O→HOO· + HO^−^ (3)

HOO· + HOO· →H_2_O_2_ + O_2_ (4)

H_2_O_2_ + e^−^ →HO· + HO^−^ (5)

O_2_·^−^/HOO·/HO· + dye → Dye degradation products (6)

Radical scavenging experiments were performed for the FcLR-gC_3_N_4_/PITN/PAN photocatalyst to identify the main reactive oxygen species (ROS) involved in the photodegradation of MO and MB dyes. BQ, TBA, and Na_2_EDTA were used as scavengers for O_2_^·–^, ·OH, and h^+^, respectively. The results showed that O_2_·^−^ and HO· are the main reactive species involved in the photocatalytic degradation of dyes.

**Figure 13:**
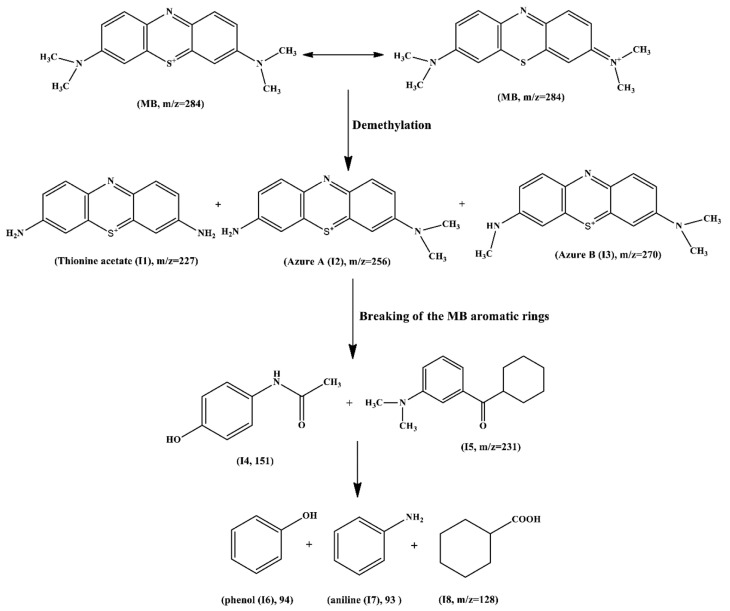
The possible intermediates generated during the photodegradation of MB. Reproduced from [93] with permission from Elsevier.

The intermediates formed during the photodegradation process of MB were quantified using a liquid chromatography–tandem mass spectrometry (LC-MS-MS) method. Figure 13 shows the main intermediates.

**Figure 14:**
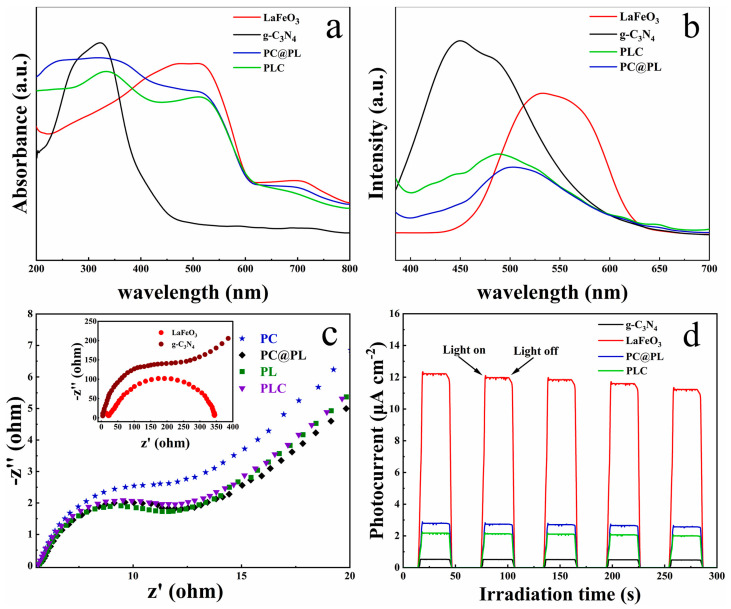
The UV–visible diffuse reflectance spectra of LaFeO_3_, g-C_3_N_4_, PC@PL, and PLC samples (**a**); the photoluminescence spectra of LaFeO_3_, g-C_3_N_4_, PC@PL, and PLC (**b**); EIS Nyquist plots of PP, PC, PL, and PC@PL (**c**); transient photocurrent response curves of LaFeO_3_, g-C_3_N_4_, and PC@PL (**d**). Reproduced from [91] with permission from Elsevier.

Mao et al. [91] developed a g-C_3_N_4_/PAN/PANI@LaFeO_3_ cable fiber membrane (PC@PL), where the spatially separated heterojunction and high conductivity of PANI enhanced carrier separation. UV–Vis diffuse reflectance spectroscopy (DRS) revealed red-shifted absorption edges for PC@PL and PLC (~660 nm) compared to pristine g-C_3_N_4_ at 480 nm—Figure 14a. PL spectra (Figure 14b) showed suppressed recombination, while EIS Nyquist plots (Figure 14c) confirmed rapid electron transfer through the PAN/PANI substrate. PANI acted as an electron mediator, further reducing recombination. Consequently, PC@PL exhibited a much higher photocurrent density than g-C_3_N_4_ and PLC (Figure 14d).

**Figure 15:**
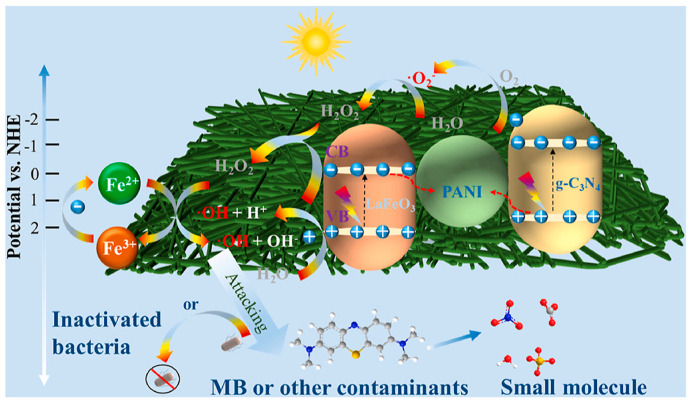
Z-scheme heterojunction structure of the g-C_3_N_4_/PAN/PANI@LaFeO_3_ (PC@PL) cable fiber membrane. Reproduced from [91] with permission from Elsevier.

A Z-scheme heterojunction structure of PC@PL cable nanofiber membranes is proposed in Figure 15.

The Z-scheme heterojunction maintained e^−^ in the CB of g-C_3_N_4_ with strong reducing ability and h^+^ in the VB of LaFeO_3_ with high oxidative competence. PANI in the cable provides abundant exposed amino/imino groups for contaminant adsorption and, due to its excellent electrical conductivity, acts as a redox mediator and adsorption matrix that facilitates e^−^/h^+^ separation from LaFeO_3_ and g-C_3_N_4_ and the degradation of pollutants via synergy between filtration, photo-Fenton catalysis, and light absorption. A PC@PL cable fiber membrane demonstrated excellent self-cleaning and photodegradation performance for MB, attaining degradation rates of 97% within 75 min under visible light and 95.8% over 180 min through static catalysis. These findings highlight that the site-specific architecture of g-C_3_N_4_ and LaFeO_3_, optimized by PANI, and the synergistic interaction between photocatalysis and filtration significantly enhanced the degradation of MB [91].

**Figure 16:**
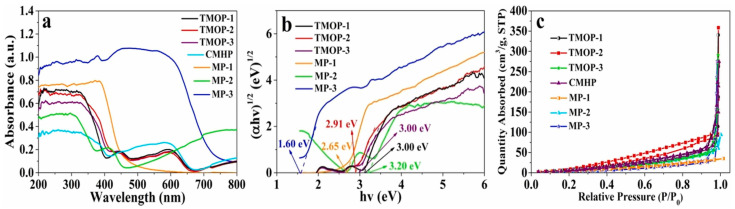
UV-Vis absorption spectra of different samples (**a**) and plots of (αhv)^1/2^ versus hv for MP-1, MP-2, and MP-3 (**b**); nitrogen adsorption–desorption isotherms of samples (**c**). Reproduced from [94] with permission from Elsevier.

Hybrid conductive nanofibers hold great potential for environmental remediation and clean energy applications by harnessing solar energy to drive processes such as the photocatalytic degradation of pollutants and solar-driven hydrogen production. Figure 16a,b illustrate the UV-Vis adsorption spectra and (αhv)^1/2^-versus-hv plots of the TMOP tricolor-typed microfiber oriented-heterostructure photocatalyst.

The calculated band-gap energies of TMOP-1, TMOP-2, and TMOP-3 were 3.11, 2.91, and 3.00 eV, respectively. The existence of micropores was proven by the nitrogen adsorption–desorption isotherms of samples—Figure 16c.

The photocatalytic performance for the removal of CIP (TMOP-1: 75.95%, TMOP-2: 88.99%, and TMOP-3: 84.69%) was higher than that of Janus microfiber photocatalysts (JMHP-1: 62.83% and JMHP-2: 54.37%).

**Figure 17:**
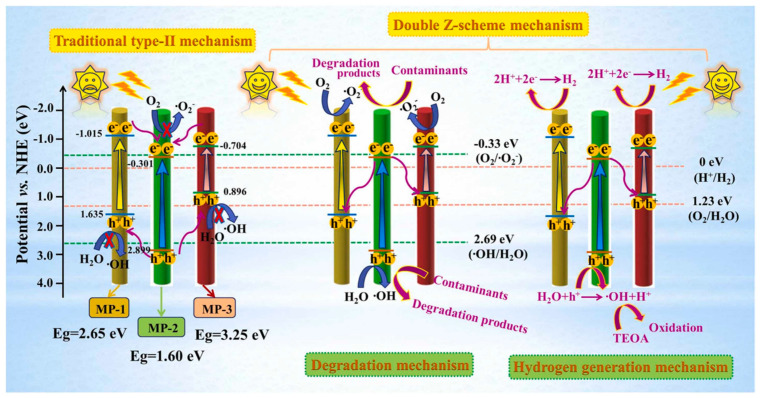
The double Z-scheme mechanism of the TMOP tricolor-typed microfiber oriented-heterostructure photocatalyst based on a synergic interaction among the three distinct microfiber components: g-C_3_N_4_/PMMA (MP-1), TiO_2_/PANI/PMMA (MP-2), and PDI/PMMA (MP-3). Reproduced from [94] with permission from Elsevier.

The mechanism of interfacial charge-carrier transfer and the improved photocatalytic performance of the TMOP tricolor-typed microfiber oriented-heterostructure photocatalyst are illustrated in Figure 17 [94].

The proposed charge transfer mechanism in TMOP followed a double Z-scheme pathway, enabled by the close interface among the three microfiber photocatalysts: MP-1 (g-C_3_N_4_/PMMA), MP-2 (TiO_2_/PANI/PMMA), and MP-3 (PDI/PMMA) [94]. Under sunlight illumination, e^−^ from the CB of MP-2 are transferred to and recombine with h^+^ in the VB of MP-1 and the HOMO of MP-3, facilitated by conductive PANI in MP-2. This process leaves behind high-energy e^−^ in the CB of MP-1 and the LUMO of MP-3 (for strong reduction) and strongly oxidative h^+^ in the VB of MP-2. This mechanism enhanced charge separation, suppressed recombination, and improved photocatalytic efficiency for the degradation of pollutants. Free radical trapping confirmed that ·O_2_^−^, ·OH, and h^+^ play key roles in TMOP-2 photocatalysis, with O_2_^−^ being the dominant species, highlighting the importance of O_2_ reduction by electrons. Additionally, the optimized TMOP-2 (containing 0.480 g TiO_2_) exhibited a high hydrogen evolution rate of 536.7 μmol·h^−1^·g^−1^, demonstrating its excellent photocatalytic efficiency for hydrogen production.

### 3.3. Schottky Junction

A Schottky junction is a type of metal–semiconductor interface that forms when a metal comes into contact with a semiconductor, resulting in a non-ohmic junction. Unlike *p*-*n* junctions formed between two semiconductors, Schottky junctions rely on the difference in work functions between the metal and the semiconductor.

**Figure 18:**
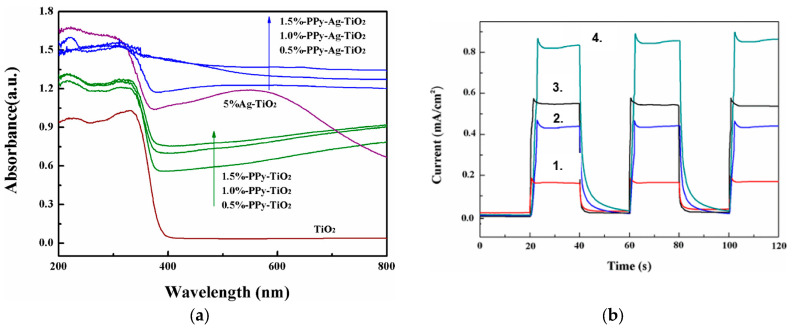
UV-vis absorption spectra of PPy-Ag-TiO_2_ with different concentrations of PPy doping (**a**); transient photocurrent responses at a constant potential of 0.5 V for (1.) TiO_2_, (2.) Ag-TiO_2_, (3.) PPy-TiO_2_, and (4.) PPy-Ag-TiO_2_ (**b**). Reproduced from [78] with permission from Elsevier.

Yang et al. [78] successfully synthesized a PPy-Ag-TiO_2_ hybrid material by decorating Ag-TiO_2_ nanofibers with a thin layer of PPy. The PPy coating effectively protected the Ag NPs from oxidative degradation. For the PPy-TiO_2_ and PPy-Ag-TiO_2_ samples, the incorporation of PPy notably influenced the light absorption of TiO_2_ and Ag-TiO_2_, with the absorption intensity increasing as the PPy doping content rose, likely due to PPy’s strong absorption across the UV and visible regions. As shown in Figure 18a, the presence of PPy in the materials is indicated by a continuous absorption band from 400 to 800 nm, with increasing absorption toward wavelengths characteristic of black solids. The absorption of PPy-Ag-TiO_2_ was significantly higher than that of PPy-TiO_2_, attributable to the presence of Ag.

Figure 18b illustrates representative photocurrent–time (I–t) response curves for the TiO_2_, Ag-TiO_2_, PPy-TiO_2_, and PPy-Ag-TiO_2_ samples during several on–off cycles of intermittent visible light illumination. The initial anodic photocurrent peak arose from electron–hole pair separation, with holes migrating to the semiconductor surface, where they were trapped or reacted with species in the electrolyte, while electrons were transported to the back contact. After this peak, the photocurrent gradually declined until a steady state was reached. The photocurrents of TiO_2_, PPy-TiO_2_, Ag-TiO_2_, and PPy-Ag-TiO_2_ electrodes were 0.18, 0.43, 0.54, and 0.83 mA/cm^2^, respectively. Ag-TiO_2_ and PPy-TiO_2_ exhibited approximately 2.38 times higher photocurrent than TiO_2_, while PPy-Ag-TiO_2_ achieved 4.6 times that of TiO_2_. The photocurrent order was PPy-Ag-TiO_2_ > PPy-TiO_2_ > Ag-TiO_2_ > TiO_2_. This marked enhancement in PPy-Ag-TiO_2_ suggests the suppressed charge recombination and more efficient separation of photogenerated electron–hole pairs at its interface.

**Figure 19:**
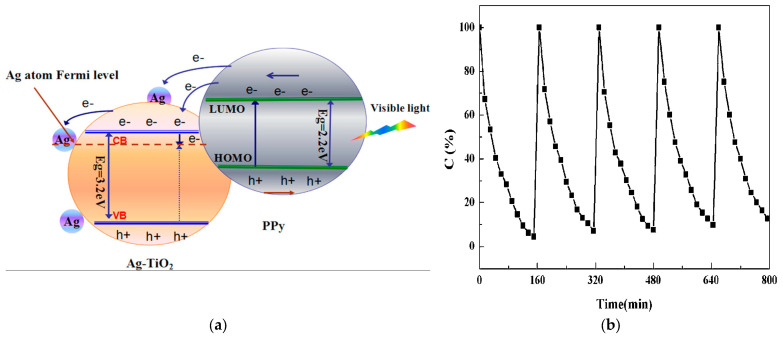
Proposed mechanism for photodegradation of acetone with PPy-Ag-TiO_2_ hybrid material (**a**); recycling of photocatalyst under visible light irradiation (**b**) [78].

Under visible light irradiation, the e^−^ in PPy were excited from the highest occupied molecular orbital (HOMO) to the lowest unoccupied molecular orbital (LUMO), leaving behind positive charge carriers (h^+^) in the HOMO (Figure 19) [78]. These photoinduced e^−^ were effectively transferred to the CB of TiO_2_ and subsequently injected into the Fermi level of Ag NPs, either directly or through the CB of TiO_2_. The interface between metallic Ag and TiO_2_ formed a Schottky junction, which acts as an efficient e^−^ trap due to the difference in work functions between Ag and TiO_2_. According to the photoluminescence (PL) emission, this junction facilitated the migration of e^−^ while suppressing their recombination with holes, thereby enhancing charge-carrier separation. The metallic Ag NPs served as e^−^ reservoirs, which promoted the reduction of dissolved oxygen. As a result, the PPy-Ag-TiO_2_ hybrid photocatalyst exhibited significantly improved photocatalytic performance for the degradation of gaseous acetone compared to its PPy-TiO_2_ counterpart. The authors observed that when the concentration of PPy surpassed 1 wt%, the surplus of PPy tended to coat the surface of Ag-TiO_2_, forming a comparatively thick coating that obstructed the migration of photoinduced e^−^ from the outer PPy layer to the inner TiO_2_ layer. Consequently, the formation of hydroxyl (·OH) radicals was diminished, adversely affecting the photodegradation efficiency of acetone. The photocatalytic activity of this PPy-Ag-TiO_2_ photocatalyst in powder form was reduced by approx. 10% after five recycling cycles in an 8 L reactor at room temperature, each with a 160 min reaction.

## 4. Challenges

Researchers have developed various methods to improve the photocatalytic performance of semiconductors for the degradation of organic contaminants in the aqueous environment. In this review, the preparation of electrospun conductive nanofibrous photocatalyst for the visible-light-driven degradation of organic contaminants in water was examined based on recently published papers. Despite significant progress in this field, several challenges and limitations remain.

Depending on the constituent materials and fabrication methods utilized, advanced electrospun composite membranes can offer a wide range of functionalities, including the degradation of organic pollutants, self-cleaning capabilities, antimicrobial effects, and improved water evaporation performance. These multifunctional properties make conductive polymers and their composites well-suited for addressing complex environmental remediation challenges. Moreover, conductive polymers can also support solar-driven hydrogen production, further expanding their application potential in sustainable energy and water treatment technologies [112]. Research has demonstrated that the incorporation of hydrophilic additives into polymer-based membranes can significantly reduce fouling while simultaneously enhancing permeability and contaminant rejection efficiency [113].

There are still relatively few studies focusing on the design of hybrid electrospun nanofibrous photocatalysts for the degradation of EOCs under visible light irradiation. Future research should explore this area more deeply. The development of highly efficient, robust, and low-cost photocatalyst systems for the degradation of organic pollutants remains a key challenge in the field of conductive electrospun photocatalysts.

Most studies report the fabrication of hybrid electrospun conductive photocatalysts by coating conductive polymers onto doped metal oxide nanofibers. In these configurations, precise control over the even distribution of the photocatalytic material on the nanofiber surface is fundamental to improving functionality. Achieving an ideal conductive polymer/semiconductor doping level that maximizes charge-carrier generation without hindering light absorption is a delicate balance.

Effective solar light absorption, photogenerated charge production, and efficient charge transfer processes are critical in determining the overall performance of conductive electrospun photocatalysts.

Globally, there is a growing recognition of the need to regulate EOCs due to their potential impact on human health and the environment. Under the Clean Water Act (CWA), the Environmental Protection Agency (EPA) sets Effluent Limitation Guidelines (ELGs) for various industries to control the discharge of pollutants into water bodies. These guidelines are technology-based and aim to protect water quality. However, many countries are still in the process of developing and implementing specific discharge limits for these contaminants. The lack of standardized regulations poses challenges for international trade and environmental protection efforts. Generally, a comparison between the fabricated photocatalysts and TiO_2_ of the gC_3_N_4_ photocatalyst is provided. Nevertheless, establishing reliable and standardized benchmarks for the photocatalytic efficiency is essential to enable meaningful comparisons across studies, guide the optimization of photocatalyst design, and ensure reproducibility under various experimental conditions.

To enable easy recovery and reuse, the photocatalyst (a combination of a conductive polymer and a semiconductor) should be immobilized onto a solid support. Particular attention must be given to the robust adhesion between immobilized catalysts and fiber substrates to prevent catalyst detachment and the resulting decline in photocatalytic performance during long-term practical operation, rather than relying solely on short-term cycling tests. The detaching of fibers during the construction of photocatalysts and their recycling cycles has led to the generation of micro- and nanoplastics. Due to their ability to adsorb other chemical pollutants in the aquatic environment, they pose a significant environmental risk, with potential health impacts on humans and aquatic organisms [6,114,115]. While photocatalysis is a promising method for degrading these particles, it is limited by incomplete mineralization and the potential formation of harmful plastic byproducts, requiring further research and optimization for effective application [116].

Many authors characterize the photocatalytic activity of conductive electrospun photocatalysts by means of photocatalytic conditions, degradation efficiency, and reusability cycles. In many cases, the complete degradation of the pollutants is not achieved, and the formation of intermediate byproducts during the degradation process is highly likely. In-depth studies are needed to investigate the formation and fate of intermediate byproducts during the photocatalytic degradation process. A few authors reported organic carbon, mineralization percent, and intermediates.

Researchers are also focusing on the development of hybrid composite nanofibers that are flexible and possess good mechanical strength. Another important issue is the stability of the polymeric substrate during repeated use of photocatalyst composites. In most laboratory-scale studies, relatively low concentrations of emerging organic pollutants (used as model contaminants) and a single pollutant have been tested. However, there remains a significant gap between laboratory results and practical, real-world applications.

It is challenging to achieve complete pollutant removal using photocatalysis alone. This indicates the need to integrate multiple treatment techniques to enhance the efficiency of emerging organic pollutant removal. Photoelectrocatalytic (PEC) membranes, which integrate photocatalytic and electrocatalytic degradation with molecular-level filtration, represent a promising approach for the rapid and efficient removal of emerging organic contaminants [54]. When an external voltage is applied, the PEC membrane (serving as the anode) facilitates e^−^ transfer from the photocatalytic layer to the conductive substrate, and then to the cathode through connecting wires. This e^−^ bypass mechanism effectively delays the recombination of photogenerated charge carriers on the anode surface, thereby addressing one of the primary limitations of conventional photocatalysis [117].

It is imperative to develop industrial photocatalysts that are both environmentally friendly and cost-effective, ensuring the simultaneous protection of the environment and conservation of energy.

Overcoming these challenges is essential for the successful deployment of photocatalytic systems in real-world environmental remediation applications. Ongoing progress in materials science, combined with interdisciplinary collaboration, holds great promise for overcoming current limitations and enabling the broader implementation of hybrid conductive electrospun photocatalysts in real-world environmental applications.

## 5. Conclusions

This review underscores the essential function of conductive polymers together with semiconductor-based photocatalysts in boosting the efficiency of composite photocatalysts for emerging organic contaminants under visible light.

Hybrid electrospun nanofibers have garnered considerable interest due to their high surface area, ease of functionalization, and enhanced charge separation efficiency, making them strong candidates for next-generation photocatalytic systems. However, the fabrication of nanofibers from conductive polymers presents certain challenges, primarily due to their rigid backbone structures and inherently low molecular weight. To overcome these limitations and improve the charge-carrier mobility of electrospun CP-based fibers, several fabrication strategies have been explored. These include (i) electrospinning or co-electrospinning of CP solutions, (ii) surface coating of non-conductive polymer fibers with conductive materials, and (iii) vacuum-assisted self-assembly techniques.

The complete degradation was not achieved in most cases, and the formation of intermediates is highly likely. The main drawbacks of the investigated hybrid electrospun conductive nanofibers in treating emerging organic contaminants are the challenges in scaling up production, ensuring the long-term stability and reusability of the catalyst, and a lack of standardized regulations for EOCs’ discharge limits and methods for evaluating performance in complex real-world wastewater matrices. Despite recent advancements, the photocatalytic activity of modified materials remains insufficient for large-scale practical applications. Emerging technologies for the rapid and efficient removal of EOCs from wastewater include photoelectrocatalytic membranes, which combine molecular-level filtration with photocatalysis and electrocatalysis.

## Figures and Tables

**Table 1:** Conductive composite fibers for organic pollutant removal from water.

Conductive Composite	Electrospinning Conditions		EOC Type	Optimized Adsorption/Photocatalysis Conditions	**Efficacity**	**Ref.**
	Processing Conditions	Solution				
**PPy-Ag-TiO_2_ nanofibers**	Voltage, flow rate, and distance from tip of needle: 10.0 kV–12.0 kV, 1 mL h^−1^, and 10.0 cm, respectively	Ag-TiO_2_ solution: 1 g of tetrabutyl titanate (TBT); 4 g of PVP, 0.1 M AgNO_3_, and 0.1 M sodium bis (2-ethylhexyl); 5 mM PPy in surfactant sodium dodecyl sulfonate (SDS)	Gaseous acetone	125 W high-pressure Hg lamp with cut-off 400 nm filter; photocatalyst powder 0.5 g; adsorption–desorption equilibrium: at 2 h in dark	Complete degradation; k = 0.087 min^−1^	[78]
**PAN@PPy–CNT(NP) mats**	For PAN mats: Voltage 16 kV; Distance from needle-collector: 20 cm; flow rate: 0.8–0.65 mL h^−1^; relative humidity: 40–48%; temperature: 22–25 °C; solution of PPY-impregnated PAN mats at 0.12 mL cm^−2^	Core solution: 20% w/w solution of PAN in DMF; shell solution: 50 mM PPY solution dissolved in 4-dodecylbenzene sulfonic acid (DBSA) in presence of 100 mM, ammonium peroxydisulfate (APS)	Model contaminants: MO (10^−5^ M), RhB (10^−5^ M), and naphthalene (5 ppm)	Sunlight simulator device with 550 W xenon lamp; irradiation time: 120 min; static conditions; adsorption–desorption equilibrium: at 2 h in dark	Adsorption efficiency of RB and naphthalene: 40 ± 10% and 35 ± 10%, respectively (2 removal cycles); TOC: 0.16 mg cm^−2^ for 60 min irradiation time	[65]
**PANI-coated TiO_2_/SiO_2_ nanofiber membranes**	Voltage, flow rate, and distance from tip of needle were: 15 kV, 2.0 mL h^−1^, and 20 cm, respectively	TiO_2_/SiO_2_ solution: 0.002 mol of TEOS and 0.01 mol of Ti(OBu)_4_; 4 wt% (PVP) in ethanol solution	MO solution (1.5 mg L^−1^)	500 W xenon lamp with cut-off 420 nm glass filter; photocatalyst membrane dimensions 1.5 cm × 0.8 cm	Degradation rate: 87% during 90 min;	[73]
**PANI@TiO_2_/PEN composite nanofibrous superhydrophilic membrane**	Voltage, flow rate, and rotational speed: 18 kV, 0.0009 mm s^−1^, and 300 rpm, respectively	2 g of PEN powder was dissolved in 5 mL of DMF solvent	MB (3 mg L^−1^)	Xenon lamp for 1 h and 1 kW m^− 2^ sunlight intensity	Degradation rate: 92.18% during 6 h	[92]
**Ferrocenyl dithiophosphonic acid (FcLR)-gC_3_N_4_/polyisothianaphthene (PITN)/PAN ternary composite nanofiber**	Voltage and flow rate: 16.5 kV and 0.5 mL h^−1^; needle diameter of 21-gauge; collector rotation speed of 100 rpm	0.3 wt% FcLR-gC_3_N_4_, 1.2 wt% PITN, and 12.0 wt% PAN were dissolved in DMF	MB (cationic dye) and MO (anionic dye), 10 mL, 5 mg L^−1^	55 W xenon lamp source, for 2 h, at room temperature and slowly stirring; photocatalytic nanofiber: 0.5 g	Degradation rates: 92% for MB and 29% for MO; MB mineralization: 65% by TOC, 60% by COD, and 55% by BOD tests after 2 h and 30 min	[93]
**MoS_2_/PANI/PAN@BiFeO_3_ bilayer hollow nanofiber membrane (PPBM-H)**	Coaxial electrospinning technique; voltage, aluminum foil collector distance, and flow were 17 kV, 15 cm, and 0.5 mL h^−1^, respectively	DMF as solvent	TCH (25 mg⋅L^−1^) and MO (15 mg⋅L^−1^) from wastewater (50 mL)	50 mg of photocatalyst was immersed in hand-made membrane filtration device containing water pump for filtering wastewater	Degradation rates: 99.5% for TCH and 99.9% for MO	[90]
**g-C_3_N_4_/PAN/PANI@LaFeO_3_ cable nanofiber membranes (PC@PL)**	Coaxial electrospinning technique; voltage, flow rate, and distance from tip of needle: 18 kV, 1.5 mL h^−1^, and 15 cm, respectively	Volume ratio of 5.068:1 between mixed solution for shell sheath and core sheath; DMF as solvent	Methyl violet (MV), CIP, and acetamiprid (AP) (all 20 mg L^−1^)	500 W spherical xenon lamp, wavelength of 300–800 nm, 30 cm distance from solution, and 37,600–39,600 Lux sunlight intensity	Degradation rates: 97.0% for MB, 94.3% for MV, 87.6% for CIP, and 88.9% for AP, within 75 min	[91]
**g-C_3_N_4_/PMMA/TiO_2_/PANI/PMMA/self-assembled 3,4,9,10-PDI/PMMA tricolor-typed microfiber oriented-heterostructure photocatalyst (TMOP)**	Voltage, flow rate, and the distance from the tip of the needle were: 10 kV, 0.8 mL h^−1^, and 15 cm; Rotation rate: 700 rpm	g-C_3_N_4_/PMMA (MP1); TiO_2_/PANI/PMMA (MP2); PDI/PMMA (MP3) (triaxial parallel electrospinning); TiO_2_ amount varied: 0.320 g, 0.480 g, and 0.640 g	CIP, TCH, chlortetracycline hydrochloride (CTC), levofloxacin (LEV), and MB	Simulated sunlight	Degradation rates: 88.99% for CIP within 90 min, 91.15% for TCH within 80 min, 77.55% for CTC within 150 min, 69.51% for LEV within 150 min, and 92.50% for MB within 50 min	[94]

**Table 2:** Properties of fabricated nanofibers and perspectives for development.

Conductive Composite	Fiber’s Characteristics	Mechanical Robustness	Recycling Cycles	Perspectives for Development	Ref.
**PPy-Ag-TiO_2_ nanofibers**	Diameter size of TiO_2_ nanofibers: ~50−200 nm; Average size of Ag NPs: 18.02 nm; Length of the fibers: a few millimeters; thickness of PPy: ~10 nm; appearance of PPy-Ag-TiO_2_ nanofibers: rather uneven	-	Photocatalytic activity decreased by ~10% after five successive reaction cycles	Promising pathway for engineering composite photocatalysts for uses beyond traditional photocatalysis	[78]
**PAN@PPy–CNT(NP) mats**	Thickness: 30 ± 10 μm; CNT (NT) detached from fibers soon after PAN@PPY–CNT(NP) mats were immersed in water	Ultimate tensile strength 10 ± 3 MPa; Elongation at break 50 ± 15%; Young’s modulus 200 ± 70 MPa (at a tensile rate of 0.01 mm s ^−1^)	Two cycles; rinsed with abundant water before subsequent cycle	Potential candidate for future large-scale photocatalytic applications	[65]
**PANI-coated TiO_2_/SiO_2_ nanofiber membranes**	Self-standing, flexible, and porous membranes (90% porosity)	-	Photocatalytic activity decreased by ~20% after five successive reaction cycles	Prospective uses in photocatalysis and aquatic pollution remediation	[73]
**PANI@TiO_2_/PEN composite nanofibrous superhydrophilic membrane**	Average diameter of bare PEN fibers: roughly 170 nm; agglomeration of PANI@TiO_2_ on PEN fibers; uniform polymerization of PANI on surface of TiO_2_ NPs; higher hydrophilicity	-	Water evaporation rates up to 3.23 kg m^−2^ h^−1^ (stability up to eight cycles); thermal and corrosion resistance	Simple and green approach to water purification	[92]
**FcLR-gC_3_N_4_/PITN/PAN ternary composite nanofiber**	Initial decomposition during temperature ranging from 295 °C to 350 °C (by TGA); higher hydrophobicity	Tensile strength: 2.17 MPa (pure PAN: 0.97 MPa); Elongation at break: 51.25% (pure PAN: 33.22%)	Good photocatalytic activity during three recycling cycles: in the case of MB, it decreased from 92% to 88%	Advanced candidate for sustainable water and wastewater treatment	[93]
**PPBM-H bilayer hollow nanofiber membrane**	Double-layer hollow structure; nanofibers are uniform without agglomeration, with rough surface; good hydrophilicity (low contact angle of 11.6°)	Young’s modulus: 81.13 MPa; Tensile strength: 2.34 MPa	After ten cycles, degradation efficiency decreased to 85.5%	Advanced self-cleaning multifunctional membranes exhibited outstanding performance, including water flux of 1248 L·m^−2^·h^−1^, BSA rejection of 98.8%, piezo-photocatalytic degradation of TCH (99.2% within 2 h), and complete disinfection of *E. coli* within 60 min	[90]
**PC@PL cable nanofiber membranes**	Average pore size of 0.92 μm; thickness of ~283 μm	Tensile strength: 2.7 MPa (meeting the requirements for filtration membranes)	After five reuse cycles, degradation efficiency was sustained at 94%	Exceptional adsorption and filtration capacity, offering practical solution for handling wastewater effluents containing wide range of contaminants such as dyes, pesticides, antibiotics, and bacteria	[91]
**TMOP tricolor-typed microfiber oriented-heterostructure photocatalyst**	Diameter sizes of photocatalyst containing different amounts of TiO_2_: 1.214 ± 0.031 µm, 1.077 ± 0.029 µm, and 1.096 ± 0.032 µm; specific surface area of 31.907 m^2^ g^−1^ for TMOP-2	-	After three consecutive cycles, photodegradation efficiency of TMOP-2 for CIP slightly decreased; stability of TMOP-2 for four reusability cycles in the case of photocatalytic hydrogen evolution	High-performance photocatalyst for concurrent removal of organic contaminants and photocatalytic hydrogen evolution; hydrogen generation efficiency: 536.7 μmol h^−1^g^−1^	[94]

## Data Availability

The data presented in this study are available on request from the corresponding authors.
